# Distance education perception scale for medical students: a validity and reliability study

**DOI:** 10.1186/s12909-021-02839-w

**Published:** 2021-07-26

**Authors:** Güven Özkaya, Mevlüt Okan Aydin, Züleyha Alper

**Affiliations:** 1 Department of Biostatistics, Faculty of Medicine, Bursa Uludağ University, Bursa, Turkey; 2 Department of Medical Education, Faculty of Medicine, Bursa Uludağ University, Bursa, Turkey

**Keywords:** Distance education, Perception, Medical students, Scale development, Validity and reliability

## Abstract

**Background:**

There are major changes in education strategies as higher education institutions urgently need to adopt distance education tools and practices due to the Covid-19 pandemic. Medical education is also trying to get out of this emergency using distance education. In this study, we aimed to develop a reliable and valid scale in order to evaluate the perceptions of medical students towards distance education.

**Methods:**

The students taking part in the study were in the first five academic years of the medical faculty in Bursa in Turkey. At first, 57 items were determined to evaluate students’ perceptions. Content validity was examined according to the assessment of the expert team. Construct validity of these items was examined by exploratory and confirmatory factor analysis. Also, Cronbach’s alpha coefficients were calculated for reliability analysis. The medical students’ responses were scored using a five-point Likert scale.

**Results:**

When the content validity was examined, the number of items was determined to be 38 items. Construct validity of these items was examined by exploratory and confirmatory factor analysis. Because of the exploratory factor analysis performed on the responses of 429 medical students, 22 items were included in four factors. This four-factor model was applied to 286 medical students and validated by confirmatory factor analysis. Also, Cronbach’s alpha coefficients were calculated for reliability analysis and values were between 0.713 and 0.930.

**Conclusions:**

This study demonstrated validation and reliability of perceptions of distance education for medical students. We suggest a 22-item model with a four-factorial scale.

## Background

In December 2020, the world was shaken by the Covid-19 pandemic. Everything we used to have lost its meaning and a new structuring was tried to be created that affected education profoundly with all its levels. It is warned that after this difficult process experienced due to the pandemic, the world may face climate change and food security problems and that there may be famine. Therefore, the economic use of resources seems to be much more important from now on. COVID-19 has led to the global testing of urgent distance education software programs. Schools, institutions, and universities are facing the challenge of how to continue teaching and learning while protecting their educators, staff, and students from the pandemic [1]. So it would be realistic to expect these systems to displace at least some of the costly face-to-face education [2].

Distance education is an educational experience in which students and educators are separated in time and place [3], and it means that education can also take place remotely at an academic institution and a degree or qualification certificate can be awarded [4]. There are different types of applications of distance education, but in this study, the assessment was conducted through online learning with synchronous, asynchronous, blended, large online open courses and open program online courses. Unlike asynchronous teaching, instructors and students meet for a session (usually online) at a predetermined time. According to Watt, live streaming video and/or audio are used for simultaneous interaction [5]. Although video-conferencing allows participants to see each other, it is not considered a face-to-face interaction due to physical separation.

With the onset of the pandemic in Turkey, asynchronous education for 1 month from March to April 2020 were initiated. In this period, educational materials were presented to students in digital media. After the learning management system infrastructure was established, the synchronous education period was started in the period of April–July 2020, during this period, education was provided online via a video-conferencing application. In July, exams were held remotely via the learning management system.

Many studies examine the perceptions of distance education according to the graduate/undergraduate students, lecturer/instructor, department/faculty/institution, courses and different countries where they study [6–13]. A large number of different studies have been conducted in which a questionnaire was applied to measure the perception of distance education among medical students [14–19]. In addition to studies using questionnaires in the evaluation of the perception of distance education, some studies develop and use scales [20–23]. Like these scales used in other students, we aim to develop a reliable and valid distance education perception scale for medical students.

## Materials and methods

### Study design and participants

This study aimed to examine the reliability and validity of the scale, that we have developed, and that we have called the “Distance Education Perception Scale - Medical Students” (DEPS - Medical Students). The subjects participating in the study consisted of medical students in the first five academic years of Bursa Uludağ University Faculty of Medicine. Bursa Uludağ University Faculty of Medicine is located in the South Marmara region which is a metropolitan area. The annual intake of the faculty is approximately 400 students. Bursa Uludağ University Pre-Graduate Medical Education program consists of three stages. Stage 1; covers the Preclinical Education-Training process, which includes 1st, 2nd, and 3rd academic year. Stage 2 includes the Clinical Education-Training process, which includes clerkship programs in 4th and 5th academic year. Stage 3 consists of 6th academic year, also known as the internship period. Medical students in 6th academic year did not participate in distance education during the internship education. They carried out the internship processes face to face by taking the necessary personal protective equipment and institutional measures. Therefore, their participation in the research was not planned. The sample size for this study was 429 medical students. Sample size calculation of studies using factor analysis in construct validity was expressed by many researchers. Some literature suggested minimum sample sizes in absolute numbers like 100–250 [24, 25], 300 [26]. In addition, Yeo and Kim [27] stated in their study that the sample size was 290 meeting the minimum requirement. They based this on the statement proposed by Kang [28] that when *n* > 200 the risk of distorting the results is low. The sample size of 429 medical students in our study meets these criteria.

### Development of the distance education perception scale for medical students (DEPS - medical students)

First, a literature review was performed by searching Turkish Index journal list (https://trdizin.gov.tr/), Google scholar, the Pubmed and Cochrane databases to define items for the first scale version. There are many studies that develop and examine the distance education perception scale [20–23]. These scales can be used for different education degrees and different departments/programs. One of these studies was remarkable. Gok and Cakmak [20] developed the distance education perception scale of faculty staff. Their study measures the distance education perceptions of lecturers at the university. They created an item pool with 82 scale items. As a result of experts’ opinions, some items were omitted from the scale and the last version of the item pool consists of 57 items. As a result of examining the reliability and validity of 57 items, they developed a scale consisting of a five-point Likert scale (between “strongly agree” and “strongly disagree”) consists of 21 items with three factors. In our study, 57 item-pool used by Gok and Cakmak [20] was taken into consideration. In these items, the statements regarding the faculty staff were adapted for the medical students. All items were positively stated. Later, it was sent to 10 experts in medical education for content validity.

The content validity of questionnaires can be examined using the assessments of the experts. The prescribed number of experts to review a scale differs from two to twenty people [29]. At least five experts were proposed to review the scale to have adequate authority over chance agreement [30]. Content validity of DEPS - Medical Students was determined using several experts (*n* = 10). The expert group of this study comprised ten senior academicians in medical education. We asked experts to rate the items in terms of their relevancy to the construct underlying study on a four-point ordinal scale (1: not relevant, 2: somewhat relevant, 3: quite relevant, 4: highly relevant). We were also asked the experts to make recommendations for improving the scale. The content validity index (CVI) is the most given index for content validity in scale development. The CVI measures the proportion of experts who are agreed on items and can be computed using Item-CVI (I-CVI). I-CVI is processed as the number of experts giving a rating of “highly relevant” for each item divided by the number of experts. I-CVI ranges from 0 to 1 where I-CVI > 0.79, the item is relevant, somewhere between 0.70 and 0.79, the item needs updates, and if I-CVI is underneath 0.70 the item is omitted [30–32].

### Data collection

Because of the pandemic, we preferred to apply the scale web-based form instead of paper-based form. We administered the scale on July 2020 after acquiring permission from the Bursa Uludağ University, Faculty of Medicine, Clinical Researches Ethics Committee (Date 10 June 2020; Number: 2020–10/25). We informed all participating medical students with the purpose and methods of the study and assured them of personal confidentiality.

### Data analysis

Descriptive statistics of demographic variables were presented by frequency and percentage. Descriptive statistics of items were given as mean and standard deviation (SD). To check for the normality of items, we calculated the skewness and kurtosis coefficients.

Exploratory factor analysis (EFA) was performed for the construct validity of the DEPS-Medical Students scale. Before examining factor structures with EFA analysis, Bartlett’s sphericity test and the Kaiser-Meyer-Olkin (KMO) measure of sampling adequacy for factor analysis were performed [33]. Thereafter, an EFA was performed with the principal components analysis (PCA) and an oblique rotation method (Promax rotation). In testing for the construct of items DEPS-Medical Students scale using EFA, we followed the four rules that are often used as the criteria for deciding about items: (i) eigenvalues larger than 1 (Kaiser criterion); (ii) factor loadings lower than < 0.40; (iii) factor loadings on over one factor; (iv) single item factors [34–36]. Confirmatory factor analysis (CFA) was also performed to investigate construct validity. It was carried out in order to measure the consistency of the rotated factor loadings structure resulting from EFA. To examine the reliability of the scale, the internal consistency reliability was used and calculated with Cronbach’s alpha coefficient. Cronbach’s α coefficients of larger than 0.70 were considered acceptable [32, 37].

EFA was performed with IBM SPSS ver.23.0 (IBM Corp. Released 2015. IBM SPSS Statistics for Windows, Version 23.0. Armonk, NY: IBM Corp.). CFA was performed using the package Lavaan 0.6–8 [38] (release date:10 March 2021) in R Studio Version 1.4.1103 (RStudio Team (2020). RStudio: Integrated Development for R. RStudio, PBC, Boston, MA URL). Statistical significance level was considered as *p* < 0.05.

## Results

A total of 429 medical students completed the web-based scale for EFA. Of the 429 medical students, 202 were male (47.1%) and 227 were female (52.9%). For CFA, 286 medical students completed the web based scale. Gender and academic year distribution of medical students were given in Table 1.

**Table 1:** Demographic variables of medical students

	EFA (***n*** = 429)	CFA (***n*** = 286)
Gender		
Female	227 (52.9%)	174 (60.8%)
Male	202 (47.1%)	112 (39.2%)
Academic year		
1st	144 (33.6%)	93 (32.5%)
2nd	97 (22.6%)	85 (29.7%)
3rd	94 (21.9%)	51 (17.8%)
4th	59 (13.8%)	38 (13.3%)
5th	35 (8.2%)	19 (6.7%)

### Content validity

The initial number of items for the DEPS - Medical Students was 57. For the content validity of the DEPS - Medical Students scale, we excluded 19 items according to assessments of the expert group because the I-CVI of items were below < 0.70. The DEPS - Medical Students scale has adequate content validity, as the remaining 38 items have I-CVI greater than 0.80.

### Exploratory factor analysis (EFA)

EFA was performed for the construct validity of the 38-item DEPS-Medical Students scale. Before using EFA, Bartlett’s sphericity test was performed with significant results (χ^2^ = 4969.778; *p* < 0.001), showing that the inter-correlation matrix contained enough common variance to make the factor analysis valuable. The KMO measure of sampling adequacy was 0.943, which suggests that these data, very suitable for factor analysis, was very sufficient. The 429 medical students’ responses were analysed using PCA and an oblique rotation method (Promax rotation). Based on previous studies, 0.40 was used in the factor loading as the cut-off score for selecting the items to be retained in the scale. Items with factor loading exceeding 0.40 and no cross-loading were assigned to factors. EFA showed that 16 items were either loaded on more than a single factor and the difference between factor loading was smaller than 0.10 or failed to load on any single factor (loading< 0.40). The retained 22-item scale was loaded on four factors (Table 2). The eigenvalues of the four factors were: λ_1_ = 9.243, λ_2_ = 1.991, λ_3_ = 1.180, λ_4_ = 1.084. The PCA findings show that the four factors with eigenvalues greater than 1.00 accounted for 61.35% of the extracted total variance in the DEPS-Medical Students scale. Description of the four factors were defined as “Students’ perception”, “Equipment facility”, “Time Management”, and “Facility and support of the institution”, respectively (Table 2). Students’ perception as Factor-I consisted of 12 items. Equipment facility as Factor-II consisted of five items. Time Management as Factor-III consisted of two items. Facility and support of the institution as Factor-IV consisted of three items.

**Table 2:** Rotated factor loadings for the 22-item DEPS - Medical Students scale

No	Item	Factors				Name of factors
		I	II	III	IV	
1	Diplomas obtained through distance education in business life are as valid as those obtained through face-to-face education.	**0.860**	0.059	−0.212	0.006	**Students’ perception**
2	Distance learning is academically more interesting than face-to-face education.	**0.831**	−0.002	0.058	−0.116	
3	The quality of education increases with distance education.	**0.810**	0.109	−0.065	−0.015	
4	Programs should be opened in different fields in distance education.	**0.804**	−0.266	0.005	0.103	
5	Distance education is essential to meet the need for trained manpower.	**0.785**	0.048	−0.058	−0.031	
6	I believe that in the future, distance education will be more preferred than traditional education.	**0.772**	−0.262	0.174	0.059	
7	Compared to face-to-face education, the cultural diversity of students in distance education is greater.	**0.765**	−0.055	− 0.166	0.023	
8	My experiences in distance education have positively changed my perspective on distance education.	**0.659**	0.156	0.173	−0.047	
9	In the distance education environment, students get the opportunity to think analytically.	**0.603**	0.109	0.169	−0.029	
10	Student self-control is high in distance education.	**0.558**	0.096	0.151	−0.019	
11	Distance education students socialize more in electronic environment.	**0.488**	0.232	−0.180	−0.063	
12	Compared to face-to-face education, distance education provides students with flexibility in terms of resource use.	**0.423**	0.005	0.396	0.069	
13	Communication tools used in distance education are technologically sufficient.	−0.164	**0.943**	0.001	−0.048	**Equipment facility**
14	Communication tools used in distance education are educationally sufficient.	0.158	**0.749**	0.044	−0.021	
15	Distance education programs are well planned in my institution.	−0.149	**0.746**	0.059	0.077	
16	The learning management system used in the presentation and execution of the courses is sufficient.	0.238	**0.640**	0.027	0.056	
17	The learning management system used in the presentation, execution and process of the courses is easy to use.	0.176	**0.423**	0.064	0.157	
18	Students spend less time in distance education than in face-to-face education.	−0.174	0.096	**0.933**	−0.078	**Time Management**
19	Compared to face-to-face education, distance education provides students with flexibility in terms of time usage.	0.064	−0.013	**0.836**	0.037	
20	Universities give students access to electronic material to support distance education	−0.039	−0.144	0.145	**0.891**	**Facility and support of the institution**
21	Universities prepare electronic materials such as e-books and e-journals to support distance education for students.	0.018	0.110	−0.198	**0.786**	
22	Students are provided with sufficient technical support to solve technical problems they encounter in distance education.	0.000	0.217	−0.022	**0.639**	
	**Eigenvalues (λ)**	9.243	1.991	1.180	1.084	

Highest factor loadings were given in bold

### Confirmatory factor analysis (CFA)

As the 22 items were loaded four factors in EFA, a CFA was performed for the other medical students (*n* = 286) resulting in same four-factor structure. When the coefficients of skewness and kurtosis were examined, it was seen that the normality of the items in the scale was suitable for CFA (Table 3). The maximum likelihood CFA was used to test the “goodness of fit” of the four-factor model. The results showed that the chi-square value of four-factor structure was significant (χ^2^ = 409.526, degree of freedom (df) =203, *p* < 0.001). Overall fit indexes also were calculated. The relative chi-square (chi-square value /df) was 2.017 (acceptable value between 1 and 5) [39, 40]. Standardized root mean square residual (SRMR) was 0.055 (acceptable value < 0.08) [39, 41]. Root mean square error of approximation (RMSEA) was 0.06 (acceptable value < 0.10) [39, 41]. Comparative Fit Index (CFI) was 0.926 (acceptable value > 0.90) [41]. Tucker-Lewis Index (TLI) was 0.916 (acceptable value > 0.90) [40]. Measures of model fit for the CFA yielded satisfactory according to acceptable values.

**Table 3:** Descriptive statistics of items for CFA and Reliability

Item no	Mean	SD	Skewness	Kurtosis	Corrected Item-Total Correlation	Cronbach’s Alpha if Item Deleted
1	2.34	1.210	0.527	−0.743	0.670	0.926
2	2.19	1.228	0.881	−0.248	0.724	0.925
3	2.12	1.154	0.862	−0.116	0.760	0.924
4	3.39	1.178	−0.689	−0.403	0.560	0.928
5	2.53	1.218	0.358	−0.908	0.681	0.926
6	2.90	1.384	0.035	− 1.313	0.625	0.927
7	2.41	1.104	0.466	−0.619	0.533	0.928
8	2.76	1.295	0.065	−1.225	0.806	0.923
9	2.54	1.140	0.365	−0.691	0.716	0.925
10	2.73	1.241	0.193	−1.072	0.655	0.926
11	2.57	1.177	0.246	−0.997	0.445	0.930
12	2.95	1.251	0.042	−1.087	0.664	0.926
13	2.78	1.155	−0.091	−1.160	0.509	0.929
14	2.61	1.132	0.146	−0.967	0.712	0.925
15	2.66	1.146	0.140	−0.928	0.476	0.929
16	2.57	1.108	0.194	−0.868	0.730	0.925
17	3.31	1.072	−0.739	− 0.170	0.572	0.928
18	3.21	1.319	−0.301	−1.181	0.464	0.930
19	3.63	1.188	−0.789	− 0.302	0.601	0.927
20	3.51	.992	−0.918	0.454	0.360	0.931
21	2.87	1.155	−0.049	−1.047	0.316	0.932
22	2.66	1.064	0.157	−0.741	0.431	0.930

### Reliability

The internal consistency reliability of 22-item DEPS - Medical Students scale and the factors were calculated by Cronbach’s alpha coefficient. Corrected item-total correlation coefficients and Cronbach’s Alpha coefficient if item deleted were at acceptable levels (Table 3). Cronbach’s alpha coefficient of DEPS - Medical Students scale was 0.930 (Table 4). Cronbach’s alpha of the four factors were 0.921 (Factor I), 0.774 (Factor II), 0.713 (Factor III), and 0.749 (Factor IV) which were also at acceptable levels.

**Table 4:** Descriptive statistics and Cronbach’s alpha values of four-factorial scale

	Factor I	Factor II	Factor III	Factor IV	Total
Number of items	12	5	3	2	22
Minimum -Maximum values	12–60	5–25	3–15	2–10	22–110
Cronbach’s alpha	0.921	0.774	0.713	0.749	0.930
Mean	31.41	13.94	6.84	9.03	61.22
Standard deviation	10.69	4.40	2.24	2.56	16.55
Skewness	0.376	−0.036	− 0.489	− 0.297	0.158
Kurtosis	−0.285	− 0.42	− 0.574	−0.027	− 0.124

## Discussion

In this study, we aimed to develop a new scale (DEPS - Medical Students) to evaluate the perceptions of distance education among medical students. This scale was needed due to the shortcomings in currently available scales developed in the past and the possibilities that have evolved with the rapid advancement of technology.

There are many articles on the perception of distance education in the literature. For example, while Fidalgo et al. [6] applied a questionnaire to the students in their study, Bagriacik [7] and Gaytan [10] evaluated the perception of distance education with qualitative data analysis in their study. In some articles, mixed research models were used in which both quantitative and qualitative data were analysed [8, 9, 11–13]. They used questionnaires to collect quantitative data in mixed research models. Evaluation of the perception of distance education in these studies mentioned, the person, department, education level, country, etc. varies for situations. Tuma et al. [14], an example of the studies conducted to evaluate the perception of distance education of medical students, applied a questionnaire to students and faculty staff in their study. Wang et al. [15], Ibrahim et al. [16], Ibrahim et al. [17], Srinivasan [18], Gismalla et al. [19] also applied a questionnaire to evaluate the perceptions of medical students about distance education. There are also studies in which scales are used instead of questionnaires in the evaluation of the perception of distance education. Bhagat et al. [21] developed a scale to evaluate the perception of distance education among Taiwanese students with different education levels (undergraduate, master, or doctoral). In addition, while Gunduz and Isman [22] developed a scale for pre-service teachers, Karaca and Yuksekdag [23] developed a scale for nursing education.

The methods used, and the participants applied in these articles differ from our article. We obtained the items from the item pool of the study of Gok and Cakmak [20]. The items in the scale developed by Gok and Cakmak [20] to measure the perception of distance education of academic staff at the university were adapted by making necessary adaptations for medical faculty students. The purpose of our reference to a study conducted with faculty members is to examine the views of students and faculty members, who are among the education stakeholders, with similar articles. In total, 715 medical students in the first five academic years were evaluated. After analysing the answers obtained from the students, sufficient evidence was found to show that the DEPS - Medical Students scale is reliable and valid.

The content validity of DEPS was supported by the assessments of the expert group. With the examination of the I-CVI index, the number of items decreased from 57 to 38. The evidence for the construct validity of DEPS was supported by EFA and CFA. As a result of EFA, 22 items created four factors: “Students’ perception,” “Equipment facility”, “Time Management” and “Facility and support of the institution”, accounting for 61.35% of the variance. Further CFA with the maximum likelihood method supported a good fit to the model, as showed by the RMSEA, SRMR, CFI, and the TLI. The χ^2^ values for the model were significant, possibly due to the large sample size. Therefore, the calculated value of χ^2^/df also showed that the fit of the model was good. Results show that the DEPS scale can be used to reflect the distance education perception of medical students and also the functioning of the four factors. The total internal consistency reliability of the DEPS - Medical Students scale is quite high. Also, each of the reliability of four factors (students’ perception, equipment facility, time management, and facility and support of the institution) is at a satisfactory level of Cronbach’s alpha.

In our study, the scale developed for medical students consisted of 22 items and 4 factors, while Bhagat et al. [21] developed a 4-factor scale (Instructor characteristics, Social presence, Instructional design, and Trust) with 16 items to evaluate the perception of distance education among Taiwanese students with different education levels (undergraduate, master, or doctoral). The distance education perception scale developed by Gok and Cakmak [20] for faculty staff consists of 3 factors (Basic perception of distance education, Access to resources, and Education planning) with 21 items. The scale developed by Gunduz and Isman [22] for pre-service teachers consists of one factor with 17 items, while the scale developed by Karaca and Yuksekdag [23] for nursing education consists of 4 factors (learning, technology, communication-evaluation, and management affect) with 16 items. We could not find any studies evaluating the distance education perception of medical students using a scale in the databases we used while conducting literature review.

### Limitation

Due to the rapid advancement of technology in terms of software and hardware in the field of communication, there is continuous development and progress in the learning tools used. This technology change is also reflected in studies related to distance education [7, 8] . As a result, students’ perceptions of distance education change dynamically. Longitudinal studies should also be conducted to determine how the students’ perceptions of distance education change. We planned to carry out these studies in future work.

## Conclusions

The findings for content and construct validity, and reliability suggest that the DEPS - Medical Students scale is a valid and reliable scale to assess the distance education perceptions provided by medical students. Also, the DEPS scale, which consists of 22 items, is easy to use. Future studies may use DEPS - Medical Students and other related scales to examine factors associated with distance education perception in medical students. We recommend further studies with larger samples and more students to validate the findings reported here. Medical schools can use DEPS to better understand their students’ perceptions of distance education. Thus, they can apply methods to encourage effective learning and further influence teaching strategies.

## Data Availability

The datasets used and/or analysed during the current study are available from the corresponding author upon reasonable request.

## Abbreviations

DEPS -Medical students: Distance Education Perception Scale - Medical Students
EFA: Exploratory Factor Analysis
CFA: Confirmatory Factor Analysis
CVI: Content validity index
I-CVI: Item-content validity index
KMO: Kaiser-Meyer-Olkin
RMSEA: Root mean square error of approximation
SRMR: Standardized root mean square residual
CFI: Comparative Fit Index
TLI: Tucker-Lewis Index
df: degree of freedom
